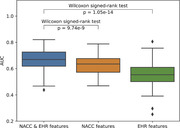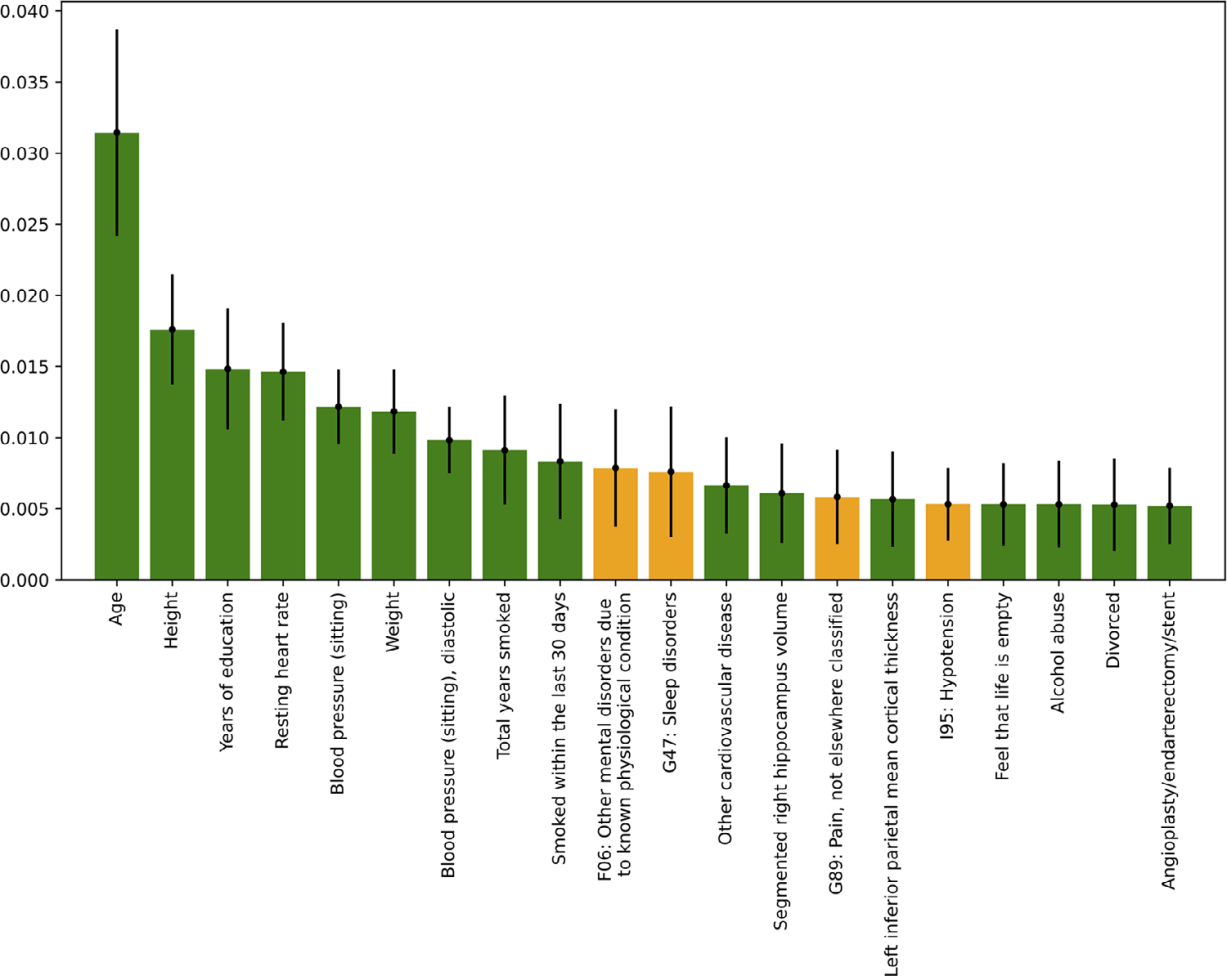# Integrating NACC UDS with RDW EHR data for MCI prediction and identification of MCI related features

**DOI:** 10.1002/alz.091635

**Published:** 2025-01-09

**Authors:** Gefei Wang, Baijia Xu, Wanwan Xu, Hiroko H Dodge, Yize Zhao

**Affiliations:** ^1^ Yale University, New Haven, CT USA; ^2^ Harvard Medical School, Boston, MA USA

## Abstract

**Background:**

Mild Cognitive Impairment (MCI) is considered as a transitional state between age‐related cognitive decline and dementia. Accurate prediction of those at risk of MCI is important for timely intervention and treatment of Alzheimer’s disease. In this study, we show that incorporating the National Alzheimer’s Coordinating Center (NACC) Uniform Data Set (UDS) with rich resources from Electronic Health Records (EHR), including comorbidities and medication histories, can achieve higher prediction accuracy, compared to using only one resource.

**Method:**

In this study, we used the Research Data Warehouse (RDW) EHR data from Oregon Health & Science University (OHSU) as auxiliary information. We first cleaned variables by rescaling and one‐hot encoding. Missing data were imputed by the average of observations. Only data recorded before MCI transition were included. To fairly compare different feature sets, the same random forest classification model with 100 estimators is used. Feature importance from the random forest identified key factors.

**Result:**

143 patients with both NACC records and OHSU RDW EHR were used after data cleaning. Among them, 85 patients transitioned to MCI. 100 random splits of training and testing datasets were generated to assess the accuracy. An Area Under the Curve (AUC) 0.671±0.075 (mean±std), sensitivity 0.759±0.102 and specificity 0.477±0.107 were achieved using variables from both NACC UDS and OHSU RDW EHR. The AUC is significantly higher than the AUC obtained only using NACC UDS (0.629±0.071) or OHSU RDW EHR(0.556±0.089), with p‐values < 0.001 using the Wilcoxon singed‐rank test (Fig. 1). The sensitivity and specificity obtained only using NACC UDS (0.726±0.108/0.457±0.109) or OHSU RDW EHR(0.687±0.126/0.333±0.140) are also relatively low. Feature importance suggested that some factors may have higher impact on MCI progression (Fig. 2). Green and orange features are from NACC UDS and OSHU RDW EHR, respectively.

**Conclusion:**

Our exploration on combining clinical variables in NACC USD with OHSU RDW EHR shows promising ability for early prediction of MCI risk. In addition to NACC UDS, hospital diagnosis histories, especially for diseases like other mental disorders, sleep disorders, pain and hypotension, provide useful information. Our study shows the potential of integrating data resources for improved prediction of MCI and identification of related factors.